# Supplementary material for: Corvids in Urban Environments: A Systematic Global Literature Review
Source: Animals (Basel). 2021 Nov 11;11(11):3226. doi: 10.3390/ani11113226 (PMC8614296; doi:10.3390/ani11113226)
Supplement: Supplementary file 1 [file animals-11-03226-s001.zip › MS_Supplementary-Material_BI et al..pdf]

Corvids in urban environments: a systematic global literature review

Isma Benmazouz 1,*, Jukka Jokimäki2, Szabolcs Lengyel3, Lajos Juhász4, Marja-Liisa Kaisanlahti-Jokimäki2, Gábor Kardos5, Petra Paládi1, and László, Kövér 4

[1] Animal Husbandry Doctoral School. University of Debrecen, Hungary; benmazouz.isma@agr.unideb.hu; paladi.petra@agr.unideb.hu
[2] Arctic Centre, University of Lapland, Finland; jukka.jokimaki@ulapland.fi; marja-liisa.kaisanlahti@ulapland.fi
[3] Department of Tisza Research, Institute of Aquatic Ecology, Centre for Ecological Research, Eötvös Loránd Research Network; lengyel.szabolcs@ecolres.hu
[4] Department of Nature Conservation Zoology and Game Management, University of Debrecen, Hungary; juhaszl@agr.unideb.hu; koverl@agr.unideb.hu
[5] Institute of Metagenomics, University of Debrecen, Hungary; kg@med.unideb.hu
* Correspondence: benmazouz.isma@agr.unideb.hu

Supplement material

Table S1: Prisma Check List.

Final search string applied to Web of Science and Scopus:

Keywords used:

"Corvids" OR "Crow" OR "jay" OR "magpie" OR "nutcracker" OR "brushcrow" OR "chough" OR "piapiac" OR "raven" OR "rook" OR "treepie" OR jackdaws" and "cities" OR "city" OR "urban" OR "suburban" OR "sub-urban" OR "town" OR "suburbs" OR "residential" OR "man-made" OR "human-made" OR "Builtup" OR "buildup" OR "built-up" OR "build-up" OR "developed" OR "non-rural" OR "metropol" AND NOT "crowd" OR "crowe" OR "crown" OR "jaya" – in topic (Title, Abstract, Key word).

Table S2: List of reviewed articles with the reference numbers used in the tables.

Table S2.xlsx

Table S3. Number of species-specific articles by species and continent (n = 424). The reference numbers in the table refers to the individual publication numbers given in the Table S2.

Table S3.docx
