# Supplementary material for: Corvids in Urban Environments: A Systematic Global Literature Review
Source: Animals (Basel). 2021 Nov 11;11(11):3226. doi: 10.3390/ani11113226 (PMC8614296; doi:10.3390/ani11113226)
Supplement: Supplementary file 1 [file animals-11-03226-s001.zip › Table S3.pdf]

Species	Continent	N	References
American Crow (*Corvus brachyrhynchos*)	America	47	40, 73, 92, 126, 154, 221, 222, 205, 91, 193, 271, 133, 134, 369, 114, 63, 74, 101, 127, 132, 150, 183, 203, 220, 235, 236, 239, 243, 244, 245, 246, 255, 268, 272, 273, 288, 351, 352, 353, 374, 381, 386, 79, 80, 81, 328, 380
Carrion Crow (*Corvus corone corone*)	Asia	8	209, 248, 388, 391, 406, 413, 415, 416
Carrion Crow (*Corvus corone corone*)	Europe	26	18, 32, 41, 94, 117, 140, 163, 164, 177, 178, 179, 189, 209, 240, 261, 295, 299, 300, 322, 323, 329, 357, 364, 371, 390, 409
Common Raven (*Corvus corax*)	America	31	92, 193, 271, 20, 37, 44, 57, 61, 75, 76, 77, 115, 147, 149, 168, 200, 201, 202, 211, 212, 232, 233, 297, 326, 333, 366, 372, 373, 376, 392, 422
Common Raven (*Corvus corax*)	Australia	1	55
Common Raven (*Corvus corax*)	Europe	15	2, 19, 26, 27, 61, 102, 163, 189, 216, 233, 242, 249, 250, 276, 397
Eurasian Magpie (*Pica pica*)	Europe	93	12, 13, 14, 18, 22, 35, 38, 41, 64, 67, 68, 71, 95, 96, 99, 100, 102, 105, 110, 113, 117, 122, 125, 128, 129, 130, 131, 135, 136, 137,138, 139, 140, 148, 157, 159, 161, 164, 169, 170, 171, 172, 176, 177, 178, 179, 180, 181, 184, 185, 186, 187, 189, 192, 197, 214, 225, 227, 234, 237, 240, 242, 249, 250, 251, 252, 270, 276, 280, 322, 325, 335, 336, 339, 340, 341, 342, 343, 344, 345, 367, 368, 382, 383, 384, 385, 393, 394, 397, 398, 408, 409, 423, 424
Eurasian Magpie (Pica pica)	Asia	1	401
Eurasian Jay (*Garrulus glandarius*)	Europe	18	41, 87, 117, 128, 131, 140, 148, 155, 177, 216, 219, 240, 242, 249, 250, 276, 335, 397
Hooded Crow (*Corvus corone cornix*)	Europe	49	23, 35, 48, 73, 97, 128, 131, 139, 157, 160, 177, 178, 179, 185, 189, 205, 209, 207, 208, 210, 216, 229, 240, 242, 249, 250, 251, 252, 256, 261, 278, 292, 307, 325, 330, 332, 335, 336, 350, 357, 364, 365, 396, 397, 398, 399, 408, 418, 423
Rook (*Corvus frugilegus*)	Europe	62	18, 32, 43, 48, 60, 71, 85, 102, 120, 140, 141, 142, 143, 144, 145, 156, 162, 165, 166, 186, 189, 198, 210, 213, 216, 230, 240, 242, 249, 250, 251, 254, 275, 276, 278, 279, 281, 282, 283, 284, 290, 292, 294, 298, 308, 311, 325, 335, 336, 355, 363, 378, 395, 397, 398, ~~402,~~ 410, 417, 418, 419, 420, 421
Rook (*Corvusfrugilegus*)	Asia	1	238
Rook (*Corvusfrugilegus*)	America	1	66
Western Jackdaw (*Coloeus monedula*)	Europe	50	11, 18, 22, 32, 33, 41, 47, 48, 60, 64, 69, 86, 102, 117, 135, 140, 144, 145, 160, 162, 177, 178, 189, 191, 216, 223, 226, 229, 240, 242, 249, 250, 251, 252, 260, 309, 310, 311, 325, 335, 336, 355, 359, 397, 398, 400, 403, 414, 420, 424

Western Jackdaw (*Coloeus monedula*)	America	2	66, 146
House Crow (*Corvus splendens*)	Asia	18	7, 16, 17, 54, 70, 106, 107, 158, 167, 182, 228, 253, 269, 289, 304, 346, 347, 348, 356, 377
House Crow (*Corvus splendens*)	Africa	6	6, 15, 121, 259, 306, 327
Jungle Crow (*Corvus macrorhynchos*)	Asia	24	17, 151, 152, 190, 204, 209, 217, 218, 248, 265, 286, 356, 370, 388, 389, 391, 404, 405, 406, 407, 411, 412, 413, 415
Scrubjays (*Aphelocoma* spp.)	America	36	3, 4, 5, 25, 40, 42, 45, 46, 49, 50, 51, 52, 53, 59, 78, 83, 90, 93, 103, 123, 153, 199, 263, 264, 266, 267, 274, 291, 312, 316, 317, 318, 319, 320, 324, 360
Blue Jay (*Cyanocitta cristata*)	America	11	28, 88, 116, 126, 221, 305, 174, 314, 331, 337, 338
Choughs (*Pyrrhocorax* spp.)	Europe	8	173, 175, 302, 303, 334, 358, 36, 285
Australian Raven (*Corvus coronoides*)	Australia	5	224, 277, 293, 296, 313
Little Raven (*Corvus mellori*)	Australia	6	30, 34, 39, 293, 362, 375
Fish Crow (*Corvus ossifragus*)	America	4	91, 154, 222, 258
Fish Crow (*Corvus ossifragus*)	America	1	247
Steller's Jay (*Cyanocitta stelleri*)	America	4	262, 301, 361, 366
Torresian Crow (*Corvus orru*)	Australia	4	55, 72, 118, 119
Korean Magpie (*Pica pica sericea*)	Asia	2	188, 196
Gray Jay (*Perisoreus canadensis*)	America	1	366
Black billed Magpie (*Pica hudsonia*)	America	1	262
Black billed Magpie (*Pica hudsonia*)	America	1	216
Chihuahuan Raven (*Corvus cryptoleucus*)	America	2	108, 109
Clark's Nutcracker (*Nucifraga columbiana*)	America	2	257, 366
Azure-winged Magpie (*Cyanopica cyanus*)	Europe	1	287
Beechey Jay (*Cyanocorax beecheii*)	America	1	379
Northwestern Crow (*Corvus caurinus*)	America	1	195
Yellow-billed Magpie (*Pica nuttalli*)	America	1	82
White-naped Jay (*Cyanocorax cyanopogon*)	America	1	21
White-necked Crow (*Corvus leucognaphalus*)	Africa	1	259
White-necked Raven (*Corvus albicollis*)	Africa	1	247
