# Supplementary figures and images for: Corvids in Urban Environments: A Systematic Global Literature Review
Source: Animals (Basel). 2021 Nov 11;11(11):3226. doi: 10.3390/ani11113226 (PMC8614296; doi:10.3390/ani11113226)

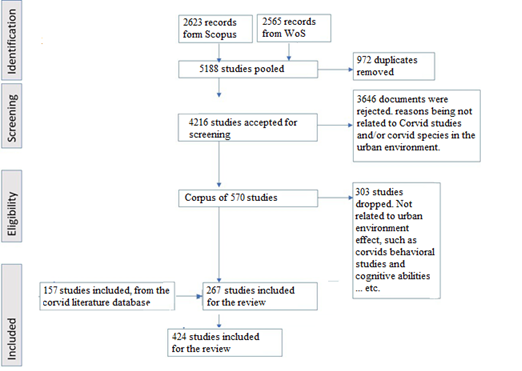

Supplement: Supplementary file 1 [file animals-11-03226-s001.zip › Table S1.png]
